# Meta-analysis of postoperative efficacy in patients receiving chemoradiotherapy followed by surgery for resectable esophageal carcinoma

**DOI:** 10.1186/1746-1596-9-151

**Published:** 2014-07-16

**Authors:** Jiaying Deng, Chunyu Wang, Mingqiong Xiang, Fatao Liu, Yun Liu, Kuaile Zhao

**Affiliations:** 1 Department of Radiation Oncology, Fudan University Shanghai Cancer Center, 270 Dongan Road, Shanghai 200032, China; 2 Shanghai Entry-Exit Inspection and Quarantine Bureau, 1208 Minsheng Road, Shanghai 200120, China; 3 Institute for Nutritional Sciences, Shanghai Institutes for Biological Sciences, Chinese Academy of Sciences, Graduate School of the Chinese Academy of Sciences, Shanghai 200031, P. R. China; 4 Institutes of Biomedical Sciences, Fudan University, Ming Dao Building, 138 Yi Xue Yuan Road, Shanghai 200032, China

**Keywords:** Esophageal carcinoma, Chemoradiotherapy followed by surgery, Surgery alone, Randomized controlled trial, Meta-analysis

## Abstract

**Background:**

Many studies have demonstrated that chemoradiotherapy followed by surgery (CRTS) prolongs the 5-year survival rate of resectable esophageal carcinoma patients. However, the effect of CRTS on postoperative complications, local recurrence and distant metastasis remains controversial. We performed a systematic review of the literature and conducted a meta-analysis to assess the postoperative efficacy of CRTS compared with surgery alone (SA).

**Methods:**

Pubmed, Web of Science and the Cochrane library Databases were used to identify published studies between 2000 and 2013 that directly compared CRTS with SA. The pooled relative risk (*RR*) and its corresponding 95% confidence interval (95% *CI*) constituted the principal measure of treatment effects. Heterogeneity was assessed by the χ^2^ and I^2^ statistic.

**Results:**

The final analysis included 1930 resectable esophageal carcinoma cases from 13 randomized controlled trials (RCTs). Compared with SA, CRTS was associated with significantly decreased postoperative mortality, local recurrence and distant metastasis rates, with *RR* (95% *CI*) = 0.64 (0.49–0.84), 0.53 (0.39–0.73), 0.82 (0.68–0.98); p = 0.001, <0.00001, =0.03, respectively. However, there was no significant difference in postoperative complication incidence between the two groups (*RR,* 1.09; 95% *CI*, 0.96–1.24; p *=* 0.18).

**Conclusions:**

CRTS significantly decreased postoperative mortality, local recurrence and distant metastasis rates compared to SA. Additionally, there were no increased postoperative complications for patients with resectable esophageal carcinoma.

**Virtual Slides:**

The virtual slide(s) for this article can be found here: http://www.diagnosticpathology.diagnomx.eu/vs/1531519216130950

## Background

Esophageal cancer is a significant health problem worldwide. There are approximately 482,300 new cases of esophageal cancer and 406, 800 deaths annually [1]. Esophagectomy is considered the gold standard treatment for patients with resectable esophageal carcinoma. However, the prognosis for patients treated with surgery alone (SA) remains poor [2]. In addition to advances in supportive care, improved and standardized surgical techniques have also contributed to an increase in the rate of curative resection [3]. Despite changes in management over the past 20 years, SA leads to relatively few long-term survivors [4]. Thus, there is interest in using combinations of chemoradiotherapy and local surgical treatment due to the high rates of locoregional and distant failure. Many studies have investigated the effect of chemoradiotherapy followed by surgery (CRTS) on the postoperative survival rate and quality of life.

There is evidence that neoadjuvant chemoradiotherapy increases the rate of complete resection [5], particularly for patients with locally advanced disease. Many randomized controlled trials (RCTs) have generated promising results with respect to 5-year survival rates. The evidence supporting the survival benefits of CRTS is clear for both esophageal squamous-cell carcinoma (SCC) and adenocarcinoma (AC) histology compared with SA. Clinicians agree there is a survival advantage associated with the CRTS regimen. However, there are different opinions on the impact of postoperative treatment effects [6,7]. There is a persistent concern that neoadjuvant treatment may cause an undesirable increase in postoperative complications.

The published meta-analyses have also failed to reach a consensus on postoperative efficacy [8-10]. Therefore, we performed a meta-analysis of prospective RCTs to examine resectable esophageal carcinoma patients treated with CRTS or SA. We compared the postoperative complications, local recurrence and distant metastasis associated with the two treatment regimens. The results of this study provide valuable evidence for clinical decision-making.

## Methods

### Identification of relevant studies

We searched PubMed, Web of Science and the Cochrane library databases to retrieve related studies published between 2000 and 2013. The search terms included “neoadjuvant therapy”, “chemoradiotherapy followed by surgery”, “chemoradiotherapy”, “surgery or operation or esophagectomy”, “esophagus or esophageal cancer or carcinoma”. To Manual searches of reference lists were also performed to ensure that no studies were missed.

### Inclusion criteria

The search results met the following inclusion criteria:(1) the study was a prospective RCT designed to compare CRTS with SA treatment of resectable esophageal cancer; (2) the assessment of the liver, kidney, heart, lung, etc. for the included patients have confirmed them to be fit for surgery; (3) the study clearly described the diagnoses and the sources of the cases and controls, the sample size, risk ratio (*RR*) and their 95% confidence interval (*CI*), or data that would allow those findings to be inferred; (4) clear follow-up censored survival, complications, number of cases for local recurrence and metastasis, and follow-up rate of > 95% in the original study.

### Data extraction and quality assessment

Two investigators (Deng, Wang) independently reviewed titles and abstracts of all citations identified by the literature search. The data were extracted by the one author. Any discrepancies were resolved by discussion among all authors, and consensus was reached. For each study, the following data were extracted: the first author of the study, the year of publication, the author’s country, the treatment regimen, tumor histology, patient outcomes including postoperative mortality, complications, recurrence and distant metastasis.

The trial validity assessment was conducted in dependently and in duplicate. A trial quality score was assigned using three aspects: blinding, randomization and handling of withdrawals and dropouts. These aspects were assessed using a scale of 1–5, according to methods previously described by Jadad et al [11].

### Statistical analysis

The analysis was performed using Review Manager Version 5.2 software and Stata Version 12.0 software. To combine results from individual trials, we used the proportion of events observed in CRTS and SA groups. The *RR* with the corresponding 95% CI was calculated for each trial using the observed proportions of events. All patient data were analyzed according to intention-to-treat principles. The overall *RR* was tested for significance using a Mantel-Haenszel *χ2* test. The outcome variables were the treatment *RR* of the postoperative mortality, complications, local recurrence and distant metastasis rates between CRTS and SA groups. The statistical significance of the summary *RR* was determined with a *Z*-test, and p < 0.05 was considered statistically significant. Subgroups of patients were assigned based on ethnicity and histology, and the groups were used to compare the impact on postoperative treatment outcomes.

### Evaluation of heterogeneity and publication bias

The heterogeneity assumption was evaluated with a chisquare-based Q-test. A p value ≥ 0.10 for the Q-test indicated a lack of heterogeneity among studies. We used a fixed effects model to calculate the total *RR* and 95% *CI*, or used a random effects model. If p < 0.1 and *I*^*2*^ > 50%, the heterogeneity was regarded as statistically significant. Egger’s test was performed to assess whether there was any publication bias due to the literature evaluated.

## Results

### Characteristics of eligible studies

**Figure 1:**
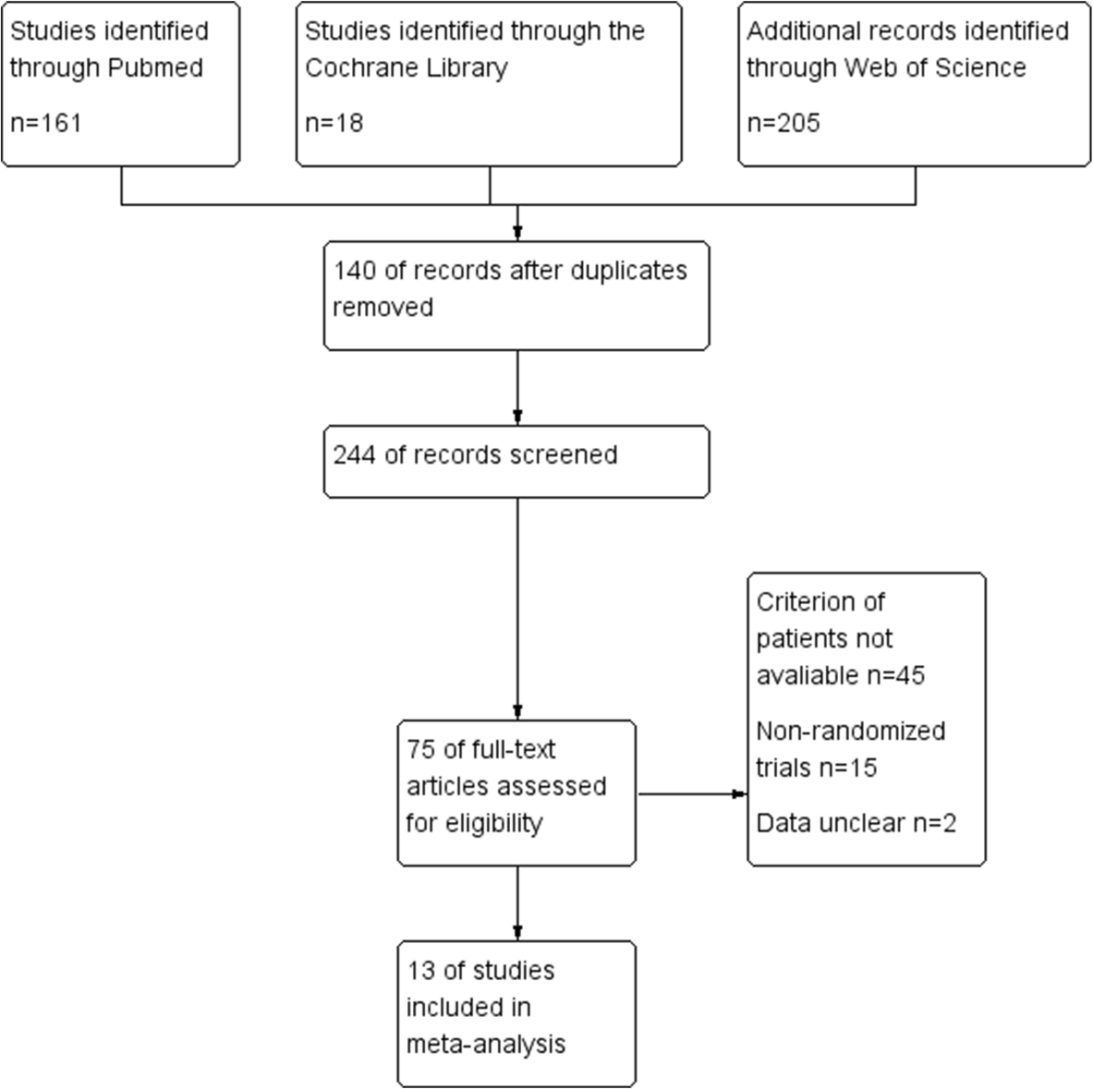
Flow chart of the meta-analysis.

We initially identified 384 relevant studies in the literature: 75 preliminary studies were included after reading the title and study summary, and 62 studies excluded after reading the full text (45 did not meet the standard of enrolled patients, 15 non-randomized controlled trials, 2 unpublished full-text articles). Thus, there were 13 RCTs that meet our inclusion criteria [12-24]. The flow chart of the meta-analysis is shown in Figure 1. There were a total of 1930 patients within these 13 RCTs. There were 970 patients in the CRTS group and 960 patients treated with SA. The selected study characteristics are summarized in Table 1.

**Table 1:** Characteristics of 13 RCTs included in the final meta-analysis

**First author**	**Year**	**Country**	**Sample size**		**Pathology**	**Neoadjuvant treatment schedule**	**Jadad score**
			CRTS	SA			
Urba [12]	2001	USA	50	50	SCC (25%); AC (75%)	45 Gy; 1.5 Gy per fraction over 3 weeks	2
						Two cycles: cisplatin 20 mg/m2 days 1–5; fluorouracil 300 mg/m2 days 1–21; vinblastine 1 mg/m2 days 1–4	
An [13]	2003	China	48	49	SCC	36 Gy, 1.2 Gy per fraction over 17 days	3
						First cycle: 5-fluorouracil 1 mg/m2, 5–6 hours, days 1–5; cisplatin 25 mg/m2.	
						Second cycle: 5-fluorouracil 0.5 g/m2, days 21–25; cisplatin 25 mg/m2, days 22–25	
Lee [14]	2004	Korea	51	50	SCC	45.6 Gy, 1.2 Gy per fraction over 28 day	2
						Two cycles: cisplatin 60 mg/m2 days 1; fluorouracil 1000 mg/m2 days 3–5	
Burmeister [15]	2005	Australia	128	128	SCC (37%); AC (62%)	35 Gy in 15 fractions over 3 weeks	3
						One cycles: cisplatin 80 mg/m2 days 1; fluorouracil 800 mg/m2 days 1–4	
Natsugoe [16]	2006	Japan	22	23	SCC	40 Gy, 2 Gy per fraction over 4 weeks	2
						cisplatin 7 mg over 2 hours; 5-fluorouracil 350 mg over 24 hours	
Tepper [17]	2008	USA	30	26	SCC (25%); AC (75%)	50.4 Gy, 1.8 Gy per fraction over 5.6 weeks	3
						Two cycles: cisplatin 100 mg/m2 days 1; fluorouracil 1000 mg/m2 per day days 1–4	
Cao [18]	2009	China	118	118	SCC	40 Gy, 2 Gy per fraction over 4 weeks	2
						cisplatin 20 mg/m2 per day days 1–5; fluorouracil 500 mg/m2 per day days 1–5; mitomycin 10 mg/m2 per day day 1	
Hurmuzlu [19]	2010	Norway	62	45	SCC (36%); AC (64%)	66 Gy, 2 Gy per fraction over 6.5 weeks	3
						Three cycles: cisplatin 100 mg/m2 days 1; fluorouracil 1000 mg/m2 per day days 1–5	
Lv [20]	2010	China	80	80	SCC	40 Gy, 2 Gy per fraction over 4 weeks	2
						Two cycles: cisplatin 20 mg/m2 per day, days 1–3, 22–25;	
						paclitaxel 135 mg/m2	
Saeki [24]	2011	Japan	76	92	SCC	30-42 Gy , 1.8 Gy per fraction	3
						cisplatin: 5 mg/m^2^/day, 5-FU: 250 mg/m^2^/day, administered on weekdays, repeated every 3-4 weeks	
van Hagen [21]	2012	Netherlands	168	186	SCC (23%); AC (75%)	41.4 Gy, 1.8 Gy per fraction over 4.6 weeks	2
						5 weeks chemotherapy: carboplatin area under curve =2 , paclitaxel 50 mg/m2 on day 1 weekly	
Fujiwara [22]	2013	Japan	52	36	SCC	40 Gy, 2 Gy per fraction over 4 weeks	3
						5-FU (500 mg/m2/day), CDDP (15-20 mg/day), days 1-5, repeated every 3 weeks	
Smit [23]	2013	Italy	75	75	SCC	41.4 Gy,1.8 Gy per fraction over 4.6 weeks	3
						5 weeks: paclitaxel (50 mg/m2), carboplatin (area under the curve = 2)	

CRTS: chemoradiotherapy followed by surgery SA: surgery alone SCC: squamous cell carcinoma AC: adenocarcinoma.

### Mortality and complications after surgery

**Figure 2:**
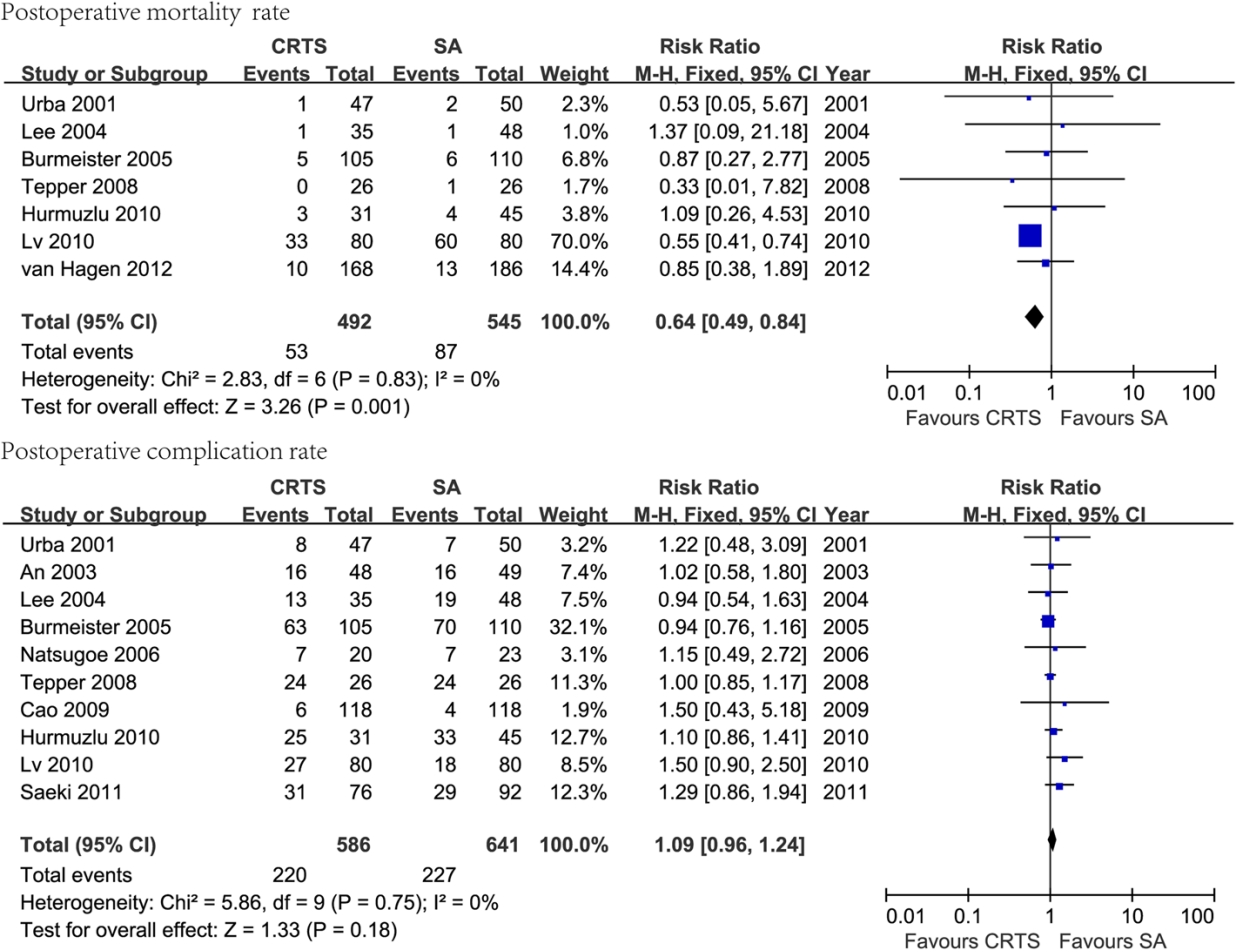
The forest plots of postoperative mortality and complication of chemoradiotherapy followed by surgery (CRTS) versus surgery alone (SA) using a fixed effects model.

The number of actual surgeries was analyzed. There was a statistically significant difference in the postoperative mortality of patients treated with CRTS compared to SA; the *RR* (95% *CI*) = 0.64 (0.49–0.84), p = 0.001. However, there was no statistical significance detected in postoperative complications; *RR* (95% *CI*) =1.09 (0.96–1.24), p *=* 0.18. All the results of the statistical analysis are shown in Table 2. The forest plots are shown in Figure 2.

**Table 2:** Meta-analysis for the outcomes of CRTS versus SA

**Outcomes**	**Included studies**	**Sample size**		**Analysis model**	**Test for overall effect**		**P value for heterogeneity**	**P value for Egger’s test**
		CRTS	SA	F	RR (95% CI)	P		
Postoperative mortality	7	492	545	F	0.64 [0.49, 0.84]	0.001	0.83	0.040
Postoperative complication	10	586	641	F	1.09 [0.96, 1.24]	0.18	0.75	0.752
Local recurrence	8	484	481	F	0.51 [0.38, 0.70]	<0.0001	0.32	0.386
Postoperative metastasis	8	484	481	F	0.82 [0.68, 0.98]	0.03	0.25	0.016

CRTS: chemoradiotherapy followed by surgery SA: surgery alone.

To examine the possible effect of ethnicity on postoperative mortality and complications, we also conducted subgroup analyses to evaluate whether patients from the West (e.g., Europe, USA) or the East (e.g., Asia) had different treatment outcomes following CRTS or SA. This subgroup analysis showed that the participant’s location had an impact on postoperative mortality for CRTS relative to SA. The RR for postoperative mortality was 0.56 (95% CI = 0.42–0.75, p < 0.0001) for the East and 0.83 (95% CI = 0.47–1.46, p = 0.52) for the West. However, the RR for postoperative complications was 1.23 (95% CI = 0.96–1.56, p = 0.10) for the East and 1 (95% CI =0.87–1.15, p = 0.98) for the West. These values were not significantly increased by CRTS (Table 3).

**Table 3:** Subgroup analysis of the postoperative treatment outcomes

**Subgroup**	**Category**	**Included studies**	**Sample size**		**Test for overall effect**	
			CRTS	SA	RR (95% CI)	*P*
Postoperative mortality based on ethnicity	The West	5	377	417	0.83 [0.47, 1.46]	0.52
	The East	2	115	128	0.56 [0.42, 0.75]	<0.0001
Postoperative complication based on ethnicity	The West	4	209	231	1.00 [0.87, 1.15]	0.98
	The East	5	357	387	1.23 [0.96, 1.56]	0.10
Local recurrence based on ethnicity	The West	4	283	279	0.51 [0.34, 0.76]	0.001
	The East	4	201	202	0.52 [0.33, 0.84]	0.008
Postoperative metastasis based on ethnicity	The West	4	283	279	0.93 [0.75, 1.16]	0.53
	The East	4	201	202	0.62 [0.44, 0.88]	0.007
Postoperative mortality based on histology	SCC	7	231	247	0.54 [0.42, 0.68]	<0.0001
	AC	5	295	313	1.26 [0.76, 2.06]	0.37

SCC: squamous cell carcinoma AC: adenocarcinoma.

A subgroup analysis by histological type for postoperative mortality where histology data were available yielded an RR for SCC of 0.54 (95% CI 0.42–0.68; p < 0.0001), in favor of CRTS. For AC, the RR was 1.26 (0.76–2.06; p = 0.37). There was a significant survival benefit for SCC, but not for AC (Table 3).

### Effect on recurrence and metastasis

**Figure 3:**
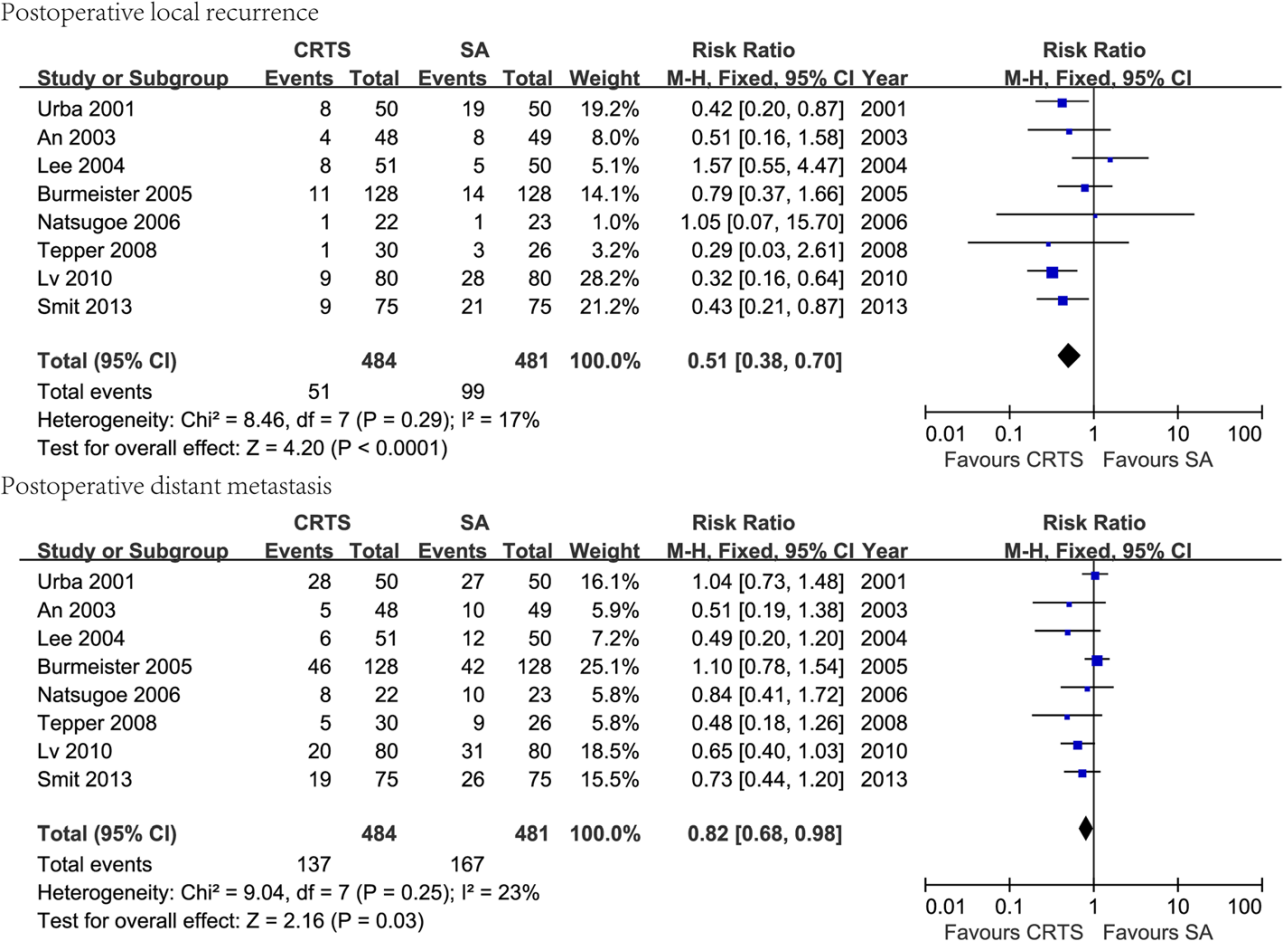
The forest plots of postoperative local recurrence and distant metastasis rates of chemoradiotherapy followed by surgery (CRTS) versus surgery alone (SA) using a fixed effects model.

Eight RCTs provided related data on tumor recurrence and metastasis. The patients treated with CRTS had markedly lower incidences of local recurrence (RR: 0.53, 95% CI: (0.39–0.73), p < 0.00001) and distant metastases (RR: 0.82, 95% CI: (0.68–0.98), p = 0.03) (Figure 3).

A subgroup analysis based on ethnicity showed that race had an impact on postoperative local recurrence and distant metastasis for CRTS relative to SA. The RR for postoperative local recurrence was 0.52 (95% CI = 0.33–0.84, p = 0.008) for the East and 0.51 (95% CI = 0.34–0.76, p = 0.001) for the West. The RR for distant metastasis for the East was 0.62 (95% CI = 0.44–0.88, p = 0.007), which was significantly increased by CRTS. However, the RR for the West was 0.93 (95% CI = 0.75–1.16, p = 0.10), which was not significantly increased by CRTS (Table 3).

### Sensitivity analysis and publication bias

The sensitivity analysis did not exclude any studies because none of the studies could alter the overall estimates for postoperative mortality, postoperative complications, postoperative local recurrence and postoperative distant metastasis. The Q-test of heterogeneity showed that these analyses had a p ≥0.10 (Table 2), which suggests that neither analyses demonstrated heterogeneity among the included RCTs. We then selected a fixed-effect model and did not introduce a subgroup analysis.

The p values based on Egger’s test for postoperative mortality, complications, local recurrence and distant metastasis are shown in Table 2. The results showed that no publication bias existed for postoperative complications and local recurrence (p *=* 0.752, 0.386, respectively). However, publication bias existed for postoperative mortality and distant metastasis (p = 0.04, 0.01, respectively). Therefore, these results need to be further examined in additional studies.

## Discussion

Previous studies have demonstrated that CRTS significantly improves the long-term survival rate of esophageal cancer compared with SA alone [25]. Consistent with this result, many RCTs and Meta studies have reached a consensus. We emphasized the investigation of neoadjuvant chemoradiotherapy in the present meta-analysis due to persistent anxiety that neoadjuvant chemoradiotherapy has a negative impact on postoperative treatment effects.

The results of our meta-analysis showed there was a statistically significant advantage for postoperative mortality, local recurrence and distant metastasis of esophageal cancer patients treated with CRTS compared with SA (p < 0.05). This finding is interestingwith respect to a recently updated meta-analysis performed by Sjoquist et al [10] published in *Lancet Oncology.* The study included 12 RCTs with 1854 cases and had strict inclusion criteria and rigorous statistical analysis. The HR for all-cause mortality for neoadjuvant chemoradiotherapy was 0.78 (95% CI 0.70-0.88; p < 0.0001). The updated meta-analysis is in accordance with our results and demonstrated the benefit of CRTS in decreasing postoperative mortality compared with SA. The survival benefits for neoadjuvant chemoradiotherapy were similar for squamous-cell carcinoma (HR 0.8, 95% CI 0.68-0.93; p = 0.004) and adenocarcinoma (HR 0.75, 0.59-0.95; p = 0.02) subgroups. The survival benefit for SCC was consistent with our subgroup analysis for SCC (RR 0.54, 95% CI 0.42–0.68; p < 0.0001). There was a smaller benefit associated with AC in our analysis, but it was not statistically significant (RR 1.26, 95% CI 0.76–2.06; p = 0.37).

In a meta-analysis conducted by K. Kumagai1 et al [26], neoadjuvant chemoradiotherapy patients with SCC were associated with a significantly higher risk of postoperative mortality (RR 1.95, 1.06 - 3.60; p **=** 0.032) compared with SA. However, there was no difference among patients with AC or in the esophageal cancer group overall. The reason for this finding may be that most of the included trials were conducted in the 1980s. After excluding trials conducted in the 1980s, the results revealed the difference was not statistically significant compared with surgery alone in the SCC subgroup.

Our meta-analysis investigated postoperative local recurrence and distant metastasis. The two indictors were rarely published in previous meta-analyses. Compared with SA, CRTS significantly decreased the local recurrence and distant metastasis rates of the tumor (p < 0.05). This finding may contribute to better survival outcomes and lower postoperative mortality. There was evidence that patients treated with SA were more likely to undergo the scheduled surgery. However, the rate of complete resection in the CRTS group was higher than in the SA group [27]. In theory, neoadjuvant chemoradiotherapy might lead to downstaging of the primary tumor and have positive effects on mediastinal nodes. This may account for the lower local recurrence rate. In this study, no subgroup analysis of postoperative complications, local recurrence or distant metastasis based on histology was investigated because the exact number of patients with these complications was not reported separately for SCC and AC.

We screened 13 RCTs based on different databases from the period of 2000 to 2013 with a larger sample size and a wider distribution range than used in previous studies. This difference is the strength of our study compared to other previous meta-analyses that included studies mostly published in 1990s. The use of radiation has developed rapidly in the past 20 years. Thus, the role of chemoradiotherapy in multimodality therapy has become more important. A systematic analysis using the latest research data is expected to produce more accurate results.

The management of esophageal cancer with neoadjuvant strategies is complex, and the available evidence is conflicting. We have discussed some of these controversies and attempted to resolve them within the context of a well-designed randomized controlled trial. We have made initial recommendations for the trial design, but this remains open for discussion and scrutiny. The meta-analysis in this study has the following limitations: some eligible studies may be missing although our big effort; the current study did lack the accurate number of patients who suffered with postoperative complication, local recurrence and distant metastasis with SCC and AC, respectively. Publication bias existed in postoperative mortality and distant metastasis, which may attribute to less studies included. It seems to us that the conclusions should be interpreted with caution. A larger number studies will be needed to verify our results further.

There is no consensus for the treatment of esophageal carcinoma and standard treatment regimens. However, most clinical studies show that preoperative chemoradiotherapy combined with surgery is a triple therapy model that may improve the clinical efficiency and the long-term survival rate. Thus, this strategy may become the standard treatment regimen [28]. A meta-analysis by Cavallin [29] revealed that patients with excellent histopathological responses benefit from neoadjuvant regimens. However, patients with poor histopathological responses have no benefit and have worse prognoses. Therefore, predictive markers to allow for individualization of multimodality treatment in locally advanced esophageal cancer are urgently needed. There was evidence that ATP-binding cassette sub-family G member 2 (ABCG2) and Vacuolar-H + -ATPase (V-ATPase) were associated with pathological grade, TNM stage and tumor metastasis in esophageal squamous cancer cells [30]. Furthermore, HER2 overexpression was associated with gastroesophageal junction (GEJ) site, intestinal cancer subtype, and well or moderately differentiated carcinomas [31]. All these markers are associated with clinicopathological features for esophageal carcinoma and contribute to optimize treatment regimen.

## Conclusions

CRTS significantly reduced postoperative mortality, local recurrence and distant metastasis rates compared to SA. However, CRTS did not increase the postoperative complication incidence compared with SA.

## Abbreviations

CRTS: Chemoradiotherapy followed by surgery; SA: Surgery alone; RCTs: Randomized controlled trials.

## Competing interests

The authors declare no competing interests.

## Authors’ contributions

KZ: conceived the study and helped to draft the manuscript; YL: checked the statistics; JD: checked the literature and performed the statistical analysis and then wrote the manuscript; CW and FL: helped with statistical analysis; MX: helped with graphs. All authors have read and approved the final manuscript.
